# The Mediating Role of Work–Family Interface in the Relationship Between Quantitative Workload and Interpersonal Strain: A Gender-Based Moderation Analysis

**DOI:** 10.3390/healthcare12232324

**Published:** 2024-11-21

**Authors:** Jessica Pileri, Marina Mondo, Alice Sgualdini, Silvia de Simone

**Affiliations:** 1 Department of Dynamic and Clinical Psychology, and Health Studies, Sapienza University of Rome, 00185 Rome, Italy; jessica.pileri@uniroma1.it; 2 Department of Pedagogy, Psychology and Philosophy, University of Cagliari, 09124 Cagliari, Italy; mmondo@unica.it; 3 Interdisciplinary Center for Gender Research and Studies, University of Cagliari, 09124 Cagliari, Italy; alice.sgualdini@unica.it

**Keywords:** work–family interface, workload, interpersonal strain, positive and negative spillovers

## Abstract

Purpose: The study investigates the role of work-family interface dimensions (negative work-to-family interface, NEGWIF; negative family-to-work interface, NEGFIW; positive work-to-family interface, POSWIF; and positive family-to-work interface, POSFIW) as mediators in the relationship between workload and interpersonal strain. In addition, we examined the moderating effect of gender. Design/methodology/approach: 319 Italian employees working in the commercial sector participants completed a self-report questionnaire. The hypothesized models were tested using PROCESS Macro. Findings: Work–family interface dimensions totally mediated the relationship between workload and interpersonal strain. Moderating influence of gender was found. Gender moderated the relationship between workload and three work–family interface dimensions—NEGWIF, POSWIF and POSFIW—in the indirect relationship between workload and interpersonal strain. Research limitations/implications: This study suggests to organizations that moderate workload and family-friendly policies can act as a protective factor against interpersonal strain. The limitations of the study are the use of self-report measures and the cross-sectional design. Originality/value: This research attempted to fill the gap in interpersonal strain and relationships with the work–family interface.

## 1. Introduction

Quality of working life encompasses a wide range of factors and is commonly described as the level of satisfaction individuals experience regarding their working conditions, the balance between work and family roles, and social dynamics within the workplace [1,2].

Recent studies have explored the connections between quality of working life and several significant constructs that influence it, including work–life balance [3,4], workload [5,6,7], and burnout [8,9]. The study of the relationships between these constructs are the basis of our research in order to identify the consequences for organizations from a gender perspective. Matters of both family and work are probably the most important issue in an individual’s life and maintaining a balance between them is closely linked to overall quality of life [10,11]. Managing these two aspects can be challenging, and the conflicting demands between the family and work domains can be correlated with depression, anxiety, and burnout [12,13]. The influence of work on private life and vice versa plays an important role in the development of burnout; specifically, previous studies attribute to the work–life interface a mediating role between the demands of the context in terms of workload and burnout [14,15,16,17,18].

As a result, extensive scientific research has focused on job burnout, analyzing both its precursors and outcomes. The phenomenon is predominantly explored through its core dimensions: exhaustion and cynicism [19,20]. Additionally, the concept of interpersonal strain [21] was introduced as a dimension capturing the interpersonal disengagement associated with burnout syndrome (ibidem). Indeed, interpersonal strain is defined as: “a specific disengagement reaction towards demanding interpersonal interactions and social pressures, through which the person creates emotional and cognitive distance from other people at work” [21] (p. 878). As Borgogni et al. [21], Consiglio [22], and Schaufeli and Enzmann [23] point out, the original interpersonal nature of burnout syndrome, which was expressed in the caregiver–recipient relationship before it was studied in all kinds of occupations, should be restored; this objective can be achieved through the study of constructs such as interpersonal strain.

Although interpersonal strain has been linked to health-related symptoms [22], it has received limited attention in prior research. Moreover, the connections between interpersonal strain and relationships outside the workplace remain underexplored. Family relationships, in particular, warrant consideration, as an increasing number of workers today must navigate the dual demands of work and family responsibilities [10,12,24,25]. The work–family interface can adversely affect health, leading to distress and significant health issues among employees [26,27,28]. This challenge may be even more pronounced for women, who often face greater difficulty in managing work and family responsibilities due to their increased involvement in family roles [29,30,31]. Evidence, including the meta-analysis by Purvanova and Muros [32] and other studies [33,34,35], suggests that women are more susceptible to symptoms of physical and emotional exhaustion. This tendency is particularly evident in Italy, the focus of our study, where traditional gender roles remain deeply entrenched, and gender disparities are significant, especially in the aftermath of the COVID-19 pandemic [36]. In Italy, women typically devote more time to unpaid domestic work than men [37], further complicating their ability to manage the work–family interface effectively.

The restrictions imposed due to COVID-19 amplified the pressures associated with both paid and unpaid workloads, as employees had to quickly adjust to remote work while managing increased household obligations [38,39]. This surge in responsibilities substantially influenced the work–family dynamic, leading to greater role strain [40,41]. Many studies noted a general rise in work–family conflict during the pandemic period [42,43]. Additionally, research highlighted that women were particularly affected, experiencing more family-to-work conflict due to elevated unpaid workload and reduced support from external services or extended family [44]. Work–family conflict during the pandemic was frequently associated with increased emotional exhaustion, stress, and burnout [43,45]. This impact was especially significant among women, who reported higher levels of physical and mental strain, as well as elevated burnout compared to men [46]. For mothers in particular, the imposed overlap of work and family responsibilities made it difficult to shift between roles, as childcare demands more acutely affected their paid work than that of fathers [47]. Violations of work-to-family boundaries were especially challenging for women, who often struggled to keep work responsibilities from interfering with family life [48]. This difficulty may stem from the fact that many women, particularly those already managing household duties, tend to place a high value on family roles and often feel they have limited control over work–family boundaries [49]. Moreover, in Italy, organizational culture is significantly shaped by a hierarchical structure characterized by centralized decision-making and a strong respect for managerial authority. A notable aspect of this culture is the emphasis on “face time”, [50] where the actual presence of employees in the workplace is often prioritized over their efficiency and productivity [51,52]. Additionally, traditional gender roles remain influential, contributing to disparities in workload and expectations for work/family responsibilities, particularly among female employees, who typically shoulder more family-related obligations. Women often bear a disproportionate share of family obligations, which is exacerbated in Italy by limited public childcare services, inadequately compensated parental leaves—primarily taken by mothers—and a scarcity of part-time job opportunities [53,54]. Social norms in Italy reinforce the perception of women as the primary caregivers [55]. These cultural characteristics are crucial to understanding how Italian employees experience and manage work–family conflict, workload, and interpersonal strain, and they are especially relevant to examining gender differences in this context. This description provides context to underscore how the unique aspects of Italian organizational culture may shape the dynamics among workload, work–family interface, and interpersonal strain.

This study aims to specifically examine interpersonal strain, the work–family interface, and workload within the theoretical framework of conservation of resources (COR) theory [56], which posits that individuals are driven to preserve and protect their resources [57]. As highlighted by Dishon-Berkovits [58] and drawing on earlier research linking the family–work interface and burnout [59], COR theory is particularly relevant to the family–work interface. On one hand, the challenges of balancing work and family roles can lead to stress stemming from resource depletion. On the other hand, positive spillovers from the work–family interface can help replenish resources, serving as a protective mechanism against burnout. Moreover, according to COR theory, threats of resource loss manifest through role demands such as a heavy workload [57], which is widely recognized as a key predictor of job burnout [60,61,62,63].

In addition, the role of interpersonal strain has been generally underexplored, yet it is gaining attention in recent literature, particularly through the examination of related individual variables such as social self-efficacy [64] and social factors like context perceptions [65]. Additionally, recent studies are focusing on burnout from a relational perspective that also encompasses work and family aspects [66].

Recent literature further confirms that, for employees, burnout often impacts both the family and work domains [67]. Some gender-related effects have also been recently examined [68], though without explicitly considering the work–family interface. This study addresses this research gap by considering interpersonal strain as an outcome variable, integrating both work–family interface aspects and gender differences, with the goal of providing a more comprehensive understanding of how workload dynamics, the work–family interface, and gender influence interpersonal strain.

In light of the above, the present study focuses on examining the influence of workload on interpersonal strain, analyzing the mediating role of the work–family interface as a pathway that could amplify or mitigate this impact. Specifically, the study investigates how the positive and negative spillovers within the work–family interface contribute differently to the dynamics between workload and interpersonal strain. Positive spillovers from work to family (POSWIF) and from family to work (POSFIW) may act as protective factors, potentially easing interpersonal strain. Conversely, negative spillovers (NEGWIF and NEGFIW) may exacerbate interpersonal strain. Additionally, this research incorporates a gender perspective, exploring whether the mediating effects of the work–family interface vary by gender. Gender roles and expectations can influence the ways individuals balance professional and family obligations, potentially leading to different levels of interpersonal strain under similar workloads. Consequently, this study aims to provide a comprehensive understanding of how workload and interpersonal stressors intersect with work–family dynamics and gender, offering insights into managing well-being in gendered organizational contexts.

## 2. Literature Review and Hypothesis

### 2.1. Work–Family Interface Between Workload and Interpersonal Strain

Interpersonal strain is a psychological distress resulting from interpersonal interactions [69] and represents the defensive reaction to the social tension inherent in work relationships that can generate a disengagement reaction [22,70]. Despite its importance, this dimension remains underexplored. Previous research has established links between interpersonal strain and the key dimensions of burnout—exhaustion and cynicism [21]—as well as its influence on turnover intentions [65]. Most studies have focused on interpersonal strain in the context of workplace relationships. For instance, Livi et al. [71] examined its mediating role between organizational socialization and organizational citizenship behaviors, while De Simone et al. [72] analyzed its connection with perceptions of workplace context. Interpersonal strain has also been investigated in emotionally intensive environments, such as healthcare settings [73,74,75], where it has been shown to negatively impact health [43,44,45], patient safety, and care quality [76,77]. However, the relationship between interpersonal strain and family dynamics remains unstudied. Similarly, no research to date has explored interpersonal strain in relation to both negative and positive spillovers. In broader work–family interface studies [78,79], research has predominantly focused on work–family conflict, defined as “a form of inter-role conflict in which the role pressures from the work and family domains are mutually incompatible” [80] (p. 77). This conflict is typically examined in two dimensions: work-to-family conflict (NEGWIF) and family-to-work conflict (NEGFIW) [81]. Conversely, the potential for rewarding, mutually beneficial relationships between work and family roles [27,82] is captured by enrichment theory [83,84]. This theory emphasizes how positive spillovers—work-to-family enrichment (POSWIF) and family-to-work enrichment (POSFIW)—can enhance job and family efficacy, thereby improving overall quality of life [83,85,86]. While studies on job burnout have considered the family–work interface, few have distinguished between NEGWIF and NEGFIW in their impact on burnout, with findings indicating associations with burnout symptoms [87,88,89,90]. From the perspective of enrichment theory, limited research suggests that positive spillovers negatively correlate with burnout [91]. Overall, achieving balance across roles appears crucial, as it is inversely related to burnout symptoms [92,93,94]. According to conservation of resources (COR) theory, individuals tend to generate and protect their amount and quality of resources [57]. Hobfoll [57] defines resources as entities centrally valued positively to obtain centrally valued ends. It is possible that the inability to meet the demands of both work and family can deprive people of resources, with emotional fallout, resulting in high levels of interpersonal strain.

Dishon-Berkovits [58] expands on this concept by examining the interplay between the work–family interface and burnout, emphasizing that managing the demands of both work and family can lead to significant stress due to the potential loss of resources. This perspective suggests that individuals must face the competing demands of their professional and personal lives, which can create stress when resources become depleted. The COR theory is particularly relevant in the context of the work–family interface for two primary reasons. First, the juggling act required to balance work and family responsibilities often results in increased stress, primarily due to resource loss. Individuals may feel overwhelmed by the need to meet obligations in both domains, leading to stress [95,96]. Second, supportive work environments and can enhance personal resources related to family and work domains [97]. Moreover, COR theory posits those threats to resource loss manifest through role demands, particularly in the context of high workload. High workload has been consistently identified as a major predictor of job burnout, as it creates additional pressures that can deplete an individual’s resources [18,98]. As individuals become increasingly burdened by work demands, the risk of experiencing interpersonal strain intensifies, further exacerbating the potential for burnout.

This is particularly significant when considering that interpersonal stress can trigger defense mechanisms in response to relational pressures [21]. Furthermore, it is essential to account for the impact of work demands. Workload is the use of psychological and physiological resources on task practice to achieve the requirements [99]. A high level of workload reduces positive spillovers [81] and increases negative spillovers [100]. Previous research show that workload is linked to job burnout [60,61,62,63,65]. A study [101] showed the mediating role of conflict and enrichment in the relationship between workload and burnout.

Therefore, based on these elements, we hypothesized that:

**H1.**  *Workload and negative spillovers (NEGWIF-NEGFIW) are positively associated with interpersonal strain.*

**H2.**  *Positive spillovers (POSWIF-POSFIW) are negatively associated with interpersonal strain.*

**H3.**  *The work–family interface dimensions (positive and negative, in both directions) mediate the relationship between workload and job strain.*

### 2.2. The Moderating Role of Gender

Societies establish norms, behaviors, values, and rights based on biological differences associated with sex [102], creating environments where distinct behaviors are expected according to assigned gender roles [103]. From an early age, individuals are exposed to and learn about these gender roles and expectations in various contexts [104]. Burnout is often perceived as a more “feminine” phenomenon [105], likely due to gender socialization processes that impose a heavy burden of role expectations and work–family reconciliation on women [106]. Research on job burnout, particularly its prototypical dimensions, frequently reports higher levels of burnout among women [107]. This may be attributed to women’s additional responsibilities in managing household tasks, childcare, and caregiving [108]. The dual demands of the work and family domains often reduce the time available for self-care, thereby negatively affecting individual well-being [62,109]. Therefore, in this study both the workload deriving from the work domain and the work–family interface are taken into consideration. We are not aware of any studies that specifically investigate interpersonal strain from this perspective.

Therefore, starting from these elements, we hypothesized that:

**H4.**  *Gender moderates the indirect effect of workload on interpersonal strain through (a) NEGWIF, (b) NEGFIW, (c) POSWIF, and (d) POSFIW. Specifically, the positive indirect effects via NEGWIF and NEGFIW are stronger for women, while the positive indirect effects via POSWIF and POSFIW are stronger for men.*

**Figure 1:**
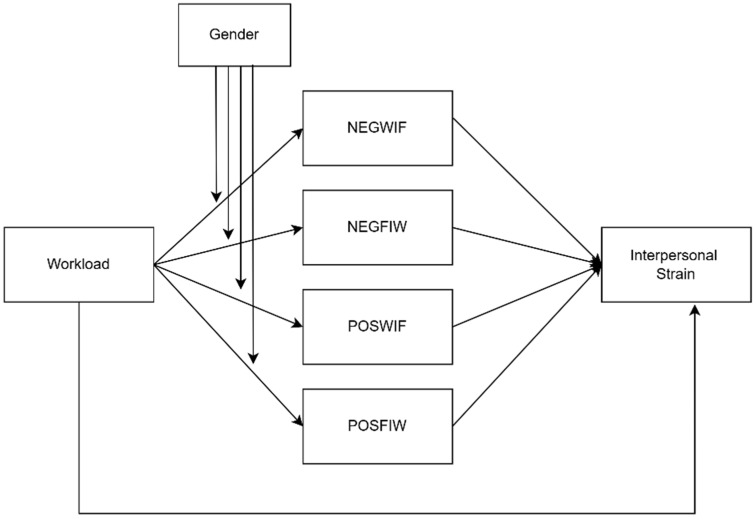
Hypothesized model.

Figure 1 illustrates the hypothesized model in the present study.

## 3. Materials and Methods

This study is based on an observational, cross-sectional design, which allowed us to collect data at a single point in time across participants.

### 3.1. Participants

The research involved a convenience sample of employees from an Italian company operating in the commercial sector. The inclusion criteria were fluency in Italian, being at least 18 years of age, and having worked in the organization for a minimum of one year. The data was produced through the on-site administration, during working hours, of a paper questionnaire. Each participant consented to take part in the study after reading an informed consent form. This form outlined the study procedures, assured privacy, and anonymity, emphasized the right to withdraw at any time, and offered an option to ask questions about the research at any stage, confirming their voluntary involvement. The sample consisted of 319 workers. In the total sample, 44.5% are men and 55.5% are women. In terms of the average age participants, it averaged at 35 years (18–64, SD = 9.45). In terms of their educational attainment, 67.7% of participants had a high-school diploma, 9,1% had completed a bachelor’s or master’s degree, and 23.1% qualified lower than diploma level. The average tenure in the organization was 5 years (SD = 5.09).

### 3.2. Materials

The instrument consisted of two sections. Section 1 included questions to gather socio-demographic data (gender, age, education level, and tenure), while Section 2 presented the measurement scales in random order.

Interpersonal strain was measured using the Interpersonal Strain at Work Scale [21], validated for the Italian context (α = 0.75). This scale comprises 6 items, including examples such as “At work, I find myself to be insensitive to other people’s problems.”

Work–family interface was assessed using the scale developed by Kinnunen and colleagues [110], as validated in Italian by De Simone et al. [111]. This tool captures both positive (POSWIF, example item: “You manage your time at home more efficiently as a result of the way you do your job?”) and negative influences (NEGWIF, example item: “The demands of your job interfere with your home and family life?”) from work to family, as well as positive (POSFIW, example item: “You manage your time at work more efficiently because at home you have to do that as well?”) and negative (NEGFIW, example item: “The demands of your family or spouse/partner interfere with your work-related activities?”) influences from family to work. The scale includes 14 items in total, with 4 items for each negative interface and 3 for each positive interface. This instrument has been validated for the Italian context (NEGWIF α = 0.86; NEGFIW α = 0.64; POSWIF α = 0.80; POSFIW α = 0.72).

Finally, to measure workload, we used the Quantitative Workload Inventory (QWI) [112], which assesses perceived workload. Translation accuracy for the Italian context was verified through back-translation. This scale includes 5 items (example item: “How often does your job require you to work very fast?”) and has shown adequate reliability (α = 0.66).

All scales used a Likert scale ranging from 0 to 6, with higher scores indicating greater levels on each dimension measured. For Cronbach’s alpha, values above 0.6 were considered acceptable [113].

### 3.3. Data Analysis

The a priori sample size analysis conducted with GPower 3.1.9.4., aimed at detecting a medium effect size (f^2^ = 0.15) [114] with a 0.05 significance level and a power of 0.95, indicated a required sample size smaller than the one ultimately achieved in this study. Missing data were handled using the regression method, assuming the data were missing at random [115]. Common method bias was checked, the measurement model was tested using confirmatory factor analysis (CFA), and descriptive statistics were conducted. Bivariate analysis was conducted to examine the associations. In order to test the main hypotheses (H1, H2 and H3), we used a PROCESS (v.3.5) macro for SPSS 25 (Model 4) created by Hayes [116]. Next, to test the moderate mediation model (H4, Model 7), we added the moderator to the model, as suggested by some authors [117,118]. We used a bootstrapping method with 5000 resamples [119].

## 4. Results

The variance inflation factors were lower than 10, so there was no multicollinearity problem [120]. Harman’s single-factor test [121] was employed to assess the potential for common method variance bias, and the results indicated that common method bias was not a concern in this study. A confirmatory factor analysis (CFA) was conducted. Different measurement models were compared. The 6-factor model proved to be the one with the best and fit indices (see Table 1), the lowest factor loading was 0.48 and all factor loadings were significantly different from zero.

Table 2 presents the mean scores, standard deviations, and zero order correlations among interpersonal strain, workload and WFI dimensions. Furthermore, it displays gender comparisons for men and women concerning the mean values of the variables considered in this study. According to the *t*-test analysis, these mean values did not significantly differ. For Pearson’s correlations, according to Cohen’s criterion [114], correlation effect sizes have been interpreted as ‘large’ (r > 0.50; Cohen’s d = 0.8 or greater), ‘medium’ (r = 0.30 to 0.49; Cohen’s d = 0.5), or ‘small’ (r = 0.10 to 0.29; Cohen’s d = 0.2). Interpersonal strain was positively associated with workload (r = 0.31 ***, medium effect size), NEGWIF (r = 0.52 ***, large effect size), and NEGFIW (r = 0.60 ***, large effect size). It was negatively associated with POSWIF (r = −0.41 ***, medium effect size) and POSFIW (r = −0.60 ***, large effect size).

The results (see Table 3) indicated that workload had a not significant direct effect on interpersonal strain (*coeff* = 0.03). Workload had a positive effect on NEGWIF (*coeff* = 0.44; *p* = 0.000) and on NEGFIW (*coeff* = 0.19; *p* = 0.000), and a negative effect on POSWIF (*coeff* = −0.22; *p* = 0.000) and on POSWIF (*coeff* = −0.57; *p* = 0.000). Interpersonal strain is predicted by NEGWIF (*coeff* = 0.22; *p* = 0.000), NEGFIW (*coeff* = 0.50; *p* = 0.000), POSWIF (*coeff* = −0.13; *p* = 0.000) and POSWIF (*coeff* = −0.27; *p* = 0.000). Table 3 (and subsequently Table 6) presents the regression coefficients and model summary information, including f^2^ values. Regarding effect size, an f^2^ value of 0.02 indicates a small effect, 0.15 a medium effect, and 0.35 a large effect [122].

The effects of indirect effects of WFI dimensions on the relationships between workload and interpersonal strain dimensions are shown in Table 4.

The bootstrapped results of indirect effects of WFI dimensions on the relationships between workload and interpersonal strain dimensions are shown in Table 4. NEGWIF, NEGFIW, POSWIF, and POSFIW totally mediated the relationship between workload and interpersonal strain.

Table 5 illustrates the confidence intervals of the direct and indirect effects. NEGWIF, NEGFIW, POSWIF, and POSFIW totally mediated the relationship between workload and interpersonal strain.

Results in Table 6 show the conditional process analysis. The interaction effect of workload and gender on the work–family interface was found to be significant for mediators: NEGWIF (*coeff* = −0.27, *p* = 0.02), POSWIF (*coeff* = −0.29, *p* = 0.02), and POSFIW (*coeff* = 0.39, *p* = 0.01).

**Figure 2:**
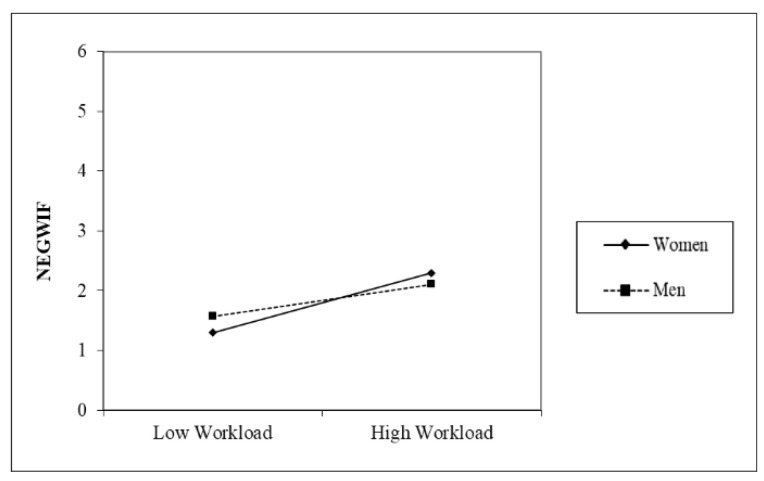
Moderating effect on NEGWIF.

**Figure 3:**
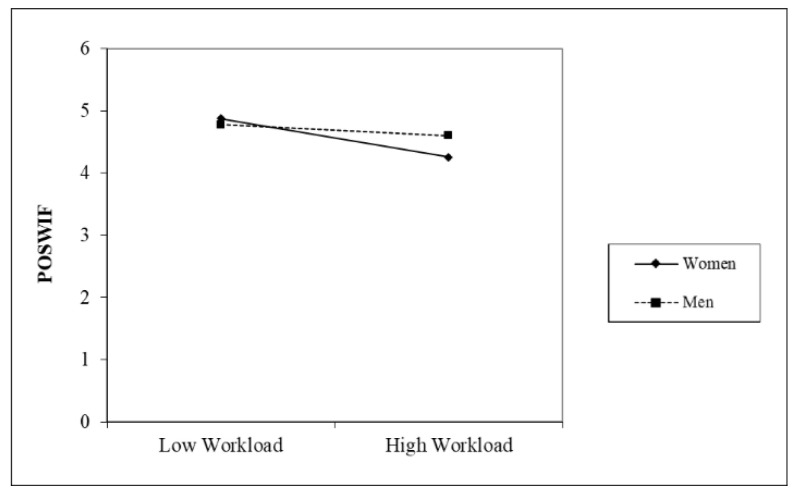
Moderating effect on POSWIF.

**Figure 4:**
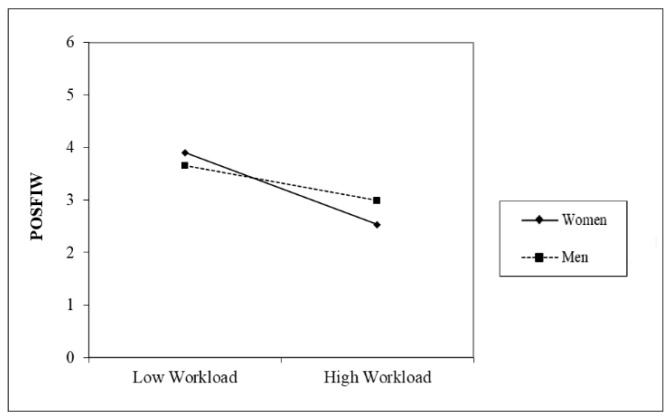
Moderating effect on POSFIW.

The moderation graphs in Figure 2, Figure 3 and Figure 4 and results show that workload was more strongly related to NEGWIF, and POSFIW for women. Furthermore, the conditional indirect effect workload → POSWIF → interpersonal strain was not significant for men.

**Figure 5:**
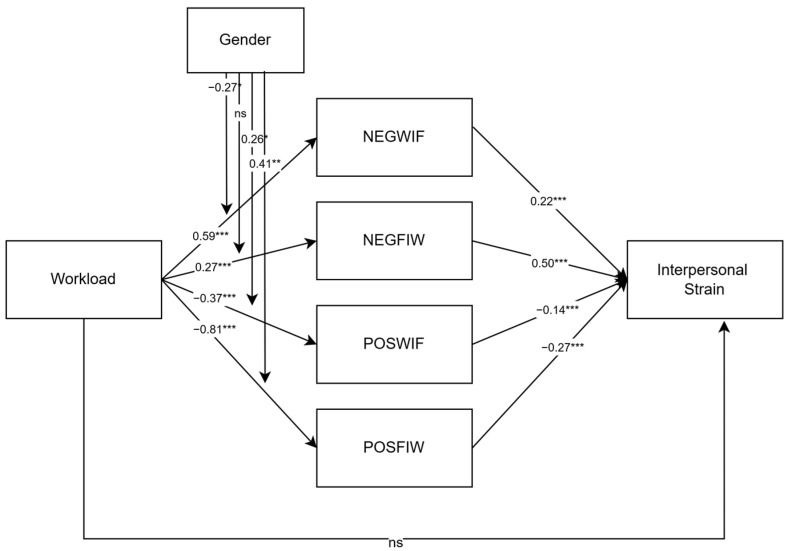
Final model. Note: values in the figure represent unstandardized coefficients. * *p* < 0.005; ** *p* < 0.01; *** *p* < 0.001. ns = not significant.

The final model is presented in Figure 5, while Table 7 offers a comprehensive overview of the hypotheses and their respective acceptance status.

## 5. Discussion

This study contributes to enriching the literature on interpersonal strain and work–family interface. Particularly, the purpose of the study was to investigate the role of the dimensions of workload, work–family interface on interpersonal strain.

H1 has been partially confirmed. The results show that both of negative dimensions of the work–family interface (NEGWIF-NEGFIW) are positively associated with interpersonal strain. As already pointed out, there is not much research on interpersonal strain. To comment on the relationship we have found between this construct and the work–family interface, we can consider the research on burnout to support our results. Several studies highlight the positive relationship between work–family conflict and burnout, pointing out that a condition of imbalance between work and life increases the risk of resource depletion [123,124,125,126,127]. According to COR theory [56,57], in the case of a conflict between work and family, people use the resources available to them to overcome this imbalance, causing a situation of emotional exhaustion such that there is a danger of disinvestment, especially at the level of relationships with colleagues and family [128].

The direct relationship between workload and interpersonal strain is, however, not significant. This result can be explained in light of the observation that interpersonal strain is a specifically relational dimension, and there is a need for the intervention of other dimensions relating to the relational aspect, in this case the work–family interface [129,130]. There may also be protective factors that have not been investigated in the research that can buffer the effects of workload on burnout (emotional exhaustion), such as self-efficacy, as shown by previous research [131,132].

H2 has been fully confirmed. The results showed that positive dimensions of the work–family interface (POSWIF-POSFIW) were negatively associated with interpersonal strain. These findings are in accordance with some of the latest research: positive work–life balance reduces burnout levels and contains the perception of loss of resources based on the characteristics and demands of the context [133,134,135]. In detail, a good work–life balance leads to an exponential increase in resources that can be invested in both areas and reach both family and work domains [83,136]. For example, this situation could also be influenced by people’s perception of being able to effectively deal with the conflict between family and work dominance. A job task successfully performed could also have positive effects in the family environment, and conversely, good management in the family environment could have positive effects in the workplace [137]. This would help and decrease the tension inherent in the working relationships that can generate a disengagement reaction [22,70].

H3 has been fully confirmed. NEGWIF, NEFIW, POSWIF and POSFIW mediated the relationship between workload and interpersonal strain. This result confirms what was said above, namely the ability of the WLB in its various positive and negative declinations to mediate the relationship between the requests of the organization that may affect the resources perceived and the negative consequences at the level of detachment in relation to interpersonal interactions and social pressures [137,138,139,140].

H4 has been partially confirmed. Gender moderated the relationship between workload and three work–family interface dimensions (NEGWIF, POSWIF and POSFIW) in the indirect relationship between workload and interpersonal strain, as confirmed by previous studies [14,15,16]. Under conditions of high workload, women suffer more negative interference from work to family (in terms of greater conflict and less enrichment), and from family to work in terms of less enrichment. Gender, however, has not moderated the relationship between workload and NEGFIW. In general, both men and women are discouraged from bringing their family problems to work.

However, there is one important aspect to be considered in understanding our findings. The organizational culture reflects the more general national culture of the country in which the organization is inserted also with reference to the many roles in which individuals are engaged [141,142,143]. For this reason, researchers prefer the development of culture-sensitive theories with constructs such as the work–family interface in order to obtain generalizable results in different cultural contents [144]. Cultures that favor a human orientation (HO) are more able to support employees in balancing work and family, a condition that is less present in Italy than in other countries [135,145]. HO refers to the presence in culture of values such as altruism, kindness, compassion, and generosity towards others; on the contrary, there are values such as self-sufficiency and a tendency to personal improvement [146,147]. Women in Italy are asked to use more resources as family support than men. Gender role theory posits that women are more inclined than men to view their family role as a fundamental component of their social identity [148]. This explains our results: in the face of increased demand at work, women are facing greater interference from work on the family, accumulating into an overload that generates a perception of scarcity of resources and indirectly affects the levels of interpersonal strain.

Our results are in line with COR theory [56,57] and enrichment theory [83,84] because where resources run out, as in the case of negative spillovers and interaction with workload, the interpersonal strain shows the highest levels; on the contrary, positive spillovers that refer to enrichment negatively affect interpersonal strain levels.

This research attempted to fill the gap in interpersonal strain and relationships with the work–family interface, but more research is needed. Future research could integrate other dimensions relating to the family and work domains and integrate a perspective that investigates adherence to gender roles. Considering the multiplicity of factors that can prevent the phenomenon of interpersonal strain, future studies could explore other dimensions that take into account the different specificities of organizations. The study is not free from limitations that may be overcome by future research, such as the type of measurement. The use of self-report questionnaires is vulnerable to prejudices of social desirability. Furthermore, the study had a cross-sectional design.

## 6. Conclusions

In conclusion, this study provides valuable theoretical and practical insights into the relationship between workload, work–family dynamics, and interpersonal strain. The findings underscore the importance of understanding these factors in both relational and organizational contexts, highlighting strategies for mitigating work–family conflict and promoting employee well-being.

Theoretically, this study contributes to the literature on interpersonal strain and the work–family interface by examining the role of workload and work–family dynamics in generating relational strain. The research highlights the importance of understanding interpersonal strain as a relational construct that is influenced not only by workload but also by positive and negative dimensions of the work–family interface. This study also emphasizes the significance of gender as a moderating factor, illustrating how the strain from work–family conflict and enrichment processes can differ based on gender roles.

Acting primarily on workload to prevent the negative spillovers of conflict from family to work and from work to family is confirmed as indispensable, as evidenced in previous research [149]. Therefore, acting on workload and creating a family-friendly organizational culture, which supports and enhances the integration between work and family life, can act as a preventive factor in relation to the onset of interpersonal strain, especially for women. From the perspective of organizations, policies that reduce conflict and promote balance between work and personal life are needed for employees.

As for the interventions that can be put in place to prevent uncomfortable conditions such as stress, burnout, and interpersonal strain, we can mention some works that can show us different ways of action. Among the most important causes of burnout and other inconveniences identified in the literature are workload, the difficulty in reconciling work and family needs, and, in general, an imbalance between demands and resources that can decrease levels of well-being and quality of working life [150,151]. To further strengthen practical applications, these implications should be delivered through specific implementation strategies, tailored to meet the needs of the corporate culture. Italian organizational culture is typically hierarchical and controlling [50,51,52]. In this context, white-collar workers should prioritize both organizational and individual interventions to effectively manage work–family integration challenges. Two types of intervention can be distinguished: those directed at individuals and those directed at organizations. Interventions directed at individuals are based on techniques to reduce the impact of imbalance, with the aim of reducing stress levels through an enhancement of self-efficacy, self-confidence, communication ability, and physical well-being [152,153,154]. Regarding clinical implications, the interesting systematic literature review by Bell et al. [155] highlights that mindfulness-based interventions may be useful, at least as a basis, though there are no clear results for burnout. Despite these outcomes, the most predominantly used interventions in research are mindfulness-based [156,157], which is considered effective for a significant decrease in burnout scores. Direct interventions in the organization include actions on workload, group, and leadership, as well as structural changes [158,159,160,161]. Other research highlights the importance of HR management interventions to a workload through three actions: effective employee selection [162,163], effective employee training [164,165], and job redesign [166,167].

## Figures and Tables

**Table 1:** Alternative measurement models on study variables.

	χ^2^	df	CFI	TLI	SRMR	RMSEA
Model 1—One Factor	1205	275	0.638	0.605	0.094	0.103
Model 2—4 Factor Model ^a^	898	269	0.755	0.727	0.097	0.086
Model 3—Complete Model	467	260	0.919	0.907	0.056	0.061

^a^ Workload, NEGWIF + NEGFIW, POSWIF + POSFIW, interpersonal strain.

**Table 2:** Descriptive statistics and correlations.

	M^tot^	M^w^	M^m^	SD	1	2	3	4	5
1. Workload	4.06	4.00	4.10	0.87	__				
2. NEGWIF	2.37	2.32	2.41	1.02	0.38 ***	__			
3. NEGFIW	0.77	0.82	0.72	0.77	0.21 ***	0.24 ***	__		
4. POSWIF	4.63	4.56	4.69	1.03	−0.19 ***	−0.30 ***	−0.29 ***	__	
5. POSFIW	3.28	3.25	3.31	1.32	−0.38 ***	−0.43 ***	−0.27 ***	0.22 ***	__
6. Interpersonal Strain	1.31	1.37	1.25	0.97	0.31 ***	0.52 ***	0.60 ***	−0.41 ***	−0.60 ***

Note. *** *p* < 0.001. M^tot^ =total mean score; M^m^ = mean men; M^w^ = mean women; SD = standard deviation.

**Table 3:** Regression coefficients and model summary information.

	M1—NEGWIF			M2—NEGFIW			M3—POSWIF			M4—POSFIW			*Y*—Interpersonal Strain		
	*coeff*	*t*	*p*	*coeff*	*t*	*p*	*coeff*	*t*	*p*	*coeff*	*t*	*p*	*coeff*	*t*	*p*
Workload	0.45	7.4	<0.001	0.19	3.9	<0.001	−0.22	−3.4	<0.001	−0.58	−7.4	<0.001	−0.03	−0.7	0.4442
NEGWIF													0.22	5.8	<0.001
NEGFIW													0.50	11.1	<0.001
POSWIF													−0.14	−4.0	<0.001
POSFIW													−0.27	−9.4	<0.001
R^2^	0.15			0.21			0.03			0.14			0.64		
*f* ^2^	0.17			0.26			0.03			0.16			1.77		

**Table 4:** Results for the indirect effects.

Model Path	Effect
Total indirect effect	0.3865
Workload → NEGWIF → Interpersonal strain	0.1003
Workload → NEGFIW → Interpersonal strain	0.0968
Workload → POSWIF → Interpersonal strain	0.0308
Workload → POSFIW → Interpersonal strain	0.1586

**Table 5:** Summary of direct and indirect effects.

Path	LLCI	ULCI
Direct effect		
Workload → NEGWIF	0.3331	0.5701
Workload → NEGFIW	0.0960	0.2867
Workload → POSWIF	−0.3522	−0.0975
Workload → POSFIW	−0.7354	−0.4285
Workload → Interpersonal strain	−0.1158	0.0509
NEGWIF → Interpersonal strain	0.1481	0.2978
NEGFIW → Interpersonal strain	0.4162	0.5969
POSWIF → Interpersonal strain	−0.2093	−0.0731
POSFIW → Interpersonal strain	−0.3330	−0.2185
Indirect effects		
	BootLLCI	BootULCI
Workload → NEGWIF → Interpersonal strain	0.0610	0.1487
Workload → NEGFIW → Interpersonal strain	0.0528	0.1440
Workload → POSWIF → Interpersonal strain	0.0107	0.0577
Workload → POSFIW → Interpersonal strain	0.1063	0.2169

Note. LLCI = lower level confidence interval; ULCI = upper level confidence interval.

**Table 6:** Conditional process analysis.

Outcome	Predictors	Coef	*p*	R^2^	*f* ^2^
NEGWIF	Workload	0.59	0.000		
	Gender	1.14	0.022		
	Int	−0.27	0.025		
				0.16	0.19
NEGFIW	Workload	0.27	0.001		
	Gender	0.44	0.265		
	Int	−0.14	0.145		
				0.05	0.05
POSWIF	Workload	−0.37	0.000		
	Gender	−0.93	0.082		
	Int	0.26	0.038		
				0.05	00.05
POSFIW	Workload	−0.81	0.000		
	Gender	−1.56	0.015		
	Int	0.41	0.007		
				0.17	00.20
**Conditional indirect effects**					
		Gender	Effect	BootLLCI	BootULCI
Workload → NEGWIF → Interpersonal strain		Women	0.1331	0.0809	0.1944
		Men	0.0729	0.0335	0.1252
Index of moderated mediation			−0.0602	−0.1170	−0.0093
Workload → NEGFIW → Interpersonal strain		Women	0.1382	0.0654	0.2199
		Men	0.0663	0.0153	0.1194
Index of moderated mediation			−0.0719	−0.1685	0.0171
Workload → POSWIF → Interpersonal strain		Women	0.0531	0.0186	0.0976
		Men	0.0152	−0.0051	0.0428
Index of moderated mediation			−0.0379	−0.0818	−0.0027
Workload → POSFIW → Interpersonal strain		Women	0.2242	0.1573	0.2983
		Men	0.1093	0.0434	0.1859
Index of moderated mediation			−0.1149	−0.2018	−0.0331

Note. LLCI = lower level confidence interval; ULCI = upper level confidence interval.

**Table 7:** Hypotheses and acceptance status.

Hypothesis	Description	Outcome	Details
H1	Workload and negative spillovers (NEGWIF-NEGFIW) are positively associated with interpersonal strain.	Partially accepted	Significant positive associations were found between NEGWIF, NEGFIW, and interpersonal strain.
H2	Positive spillovers (POSWIF-POSFIW) are negatively associated with interpersonal strain.	Accepted	Significant negative associations were found between POSWIF, POSFIW, and interpersonal strain.
H3	The work–family interface dimensions (positive and negative, in both directions) mediate the relationship between workload and interpersonal strain.	Accepted	Mediation effects were confirmed for workload on interpersonal strain through all work–family interface dimensions.
H4	Gender moderates the indirect effect of workload on interpersonal strain through (a) NEGWIF, (b) NEGFIW, (c) POSWIF, and (d) POSFIW. Specifically, positive indirect effects via NEGWIF and NEGFIW are stronger for women, while positive indirect effects via POSWIF and POSFIW are stronger for men.	Partially accepted	Gender moderation was found only for NEGWIF, POSWIF and POSFIW. No significant moderation effect was found for the relationship between workload and NEGFIW.

## Data Availability

The data supporting the conclusions of this article will be made available by the authors on reasonable request from the corresponding author.
